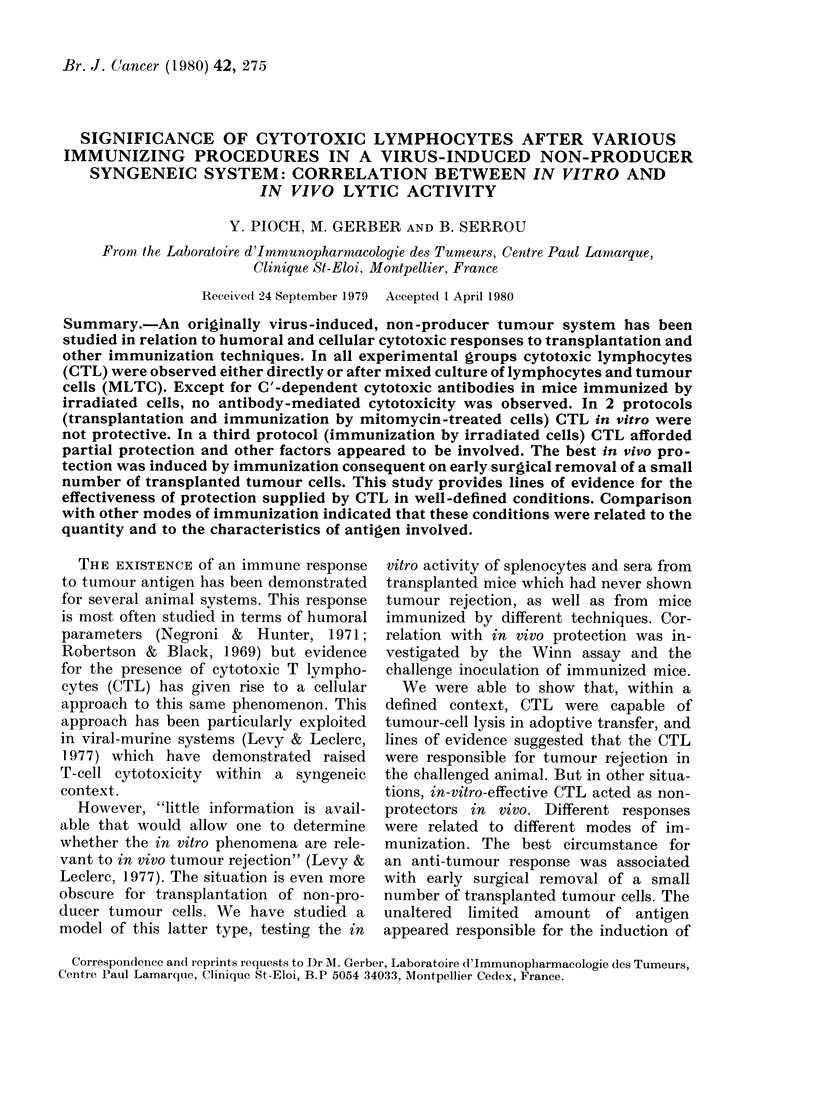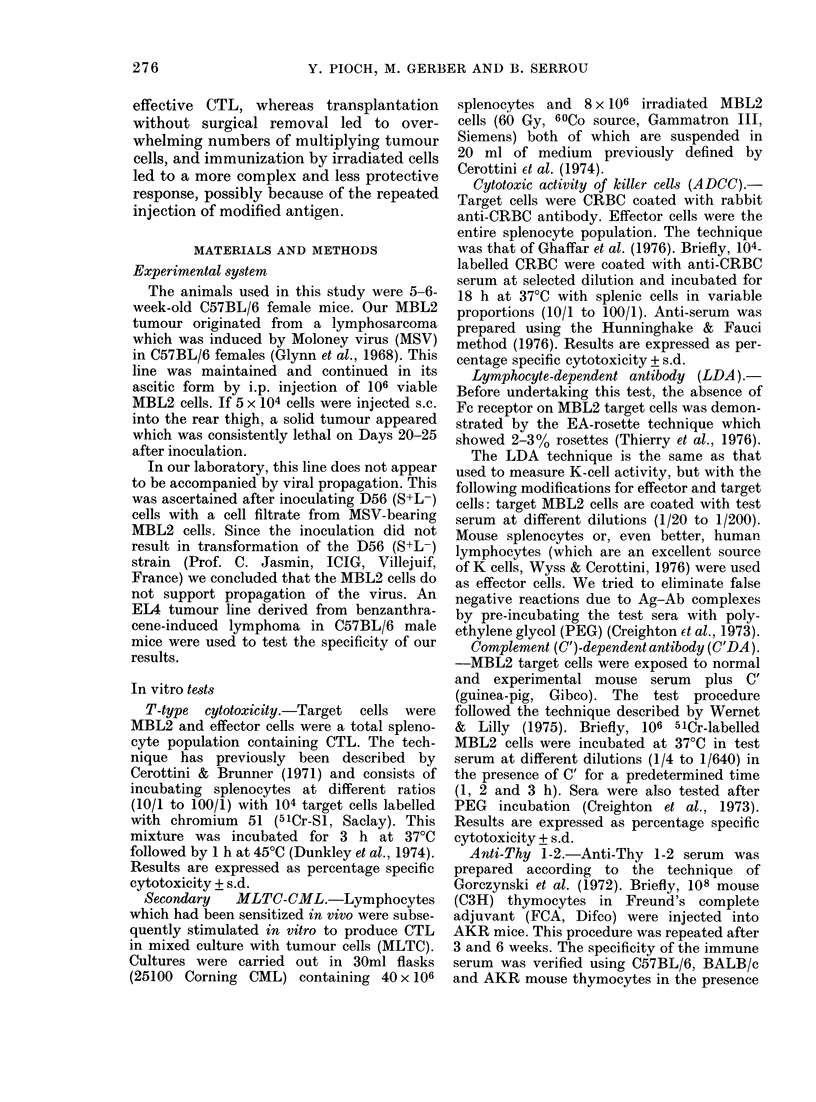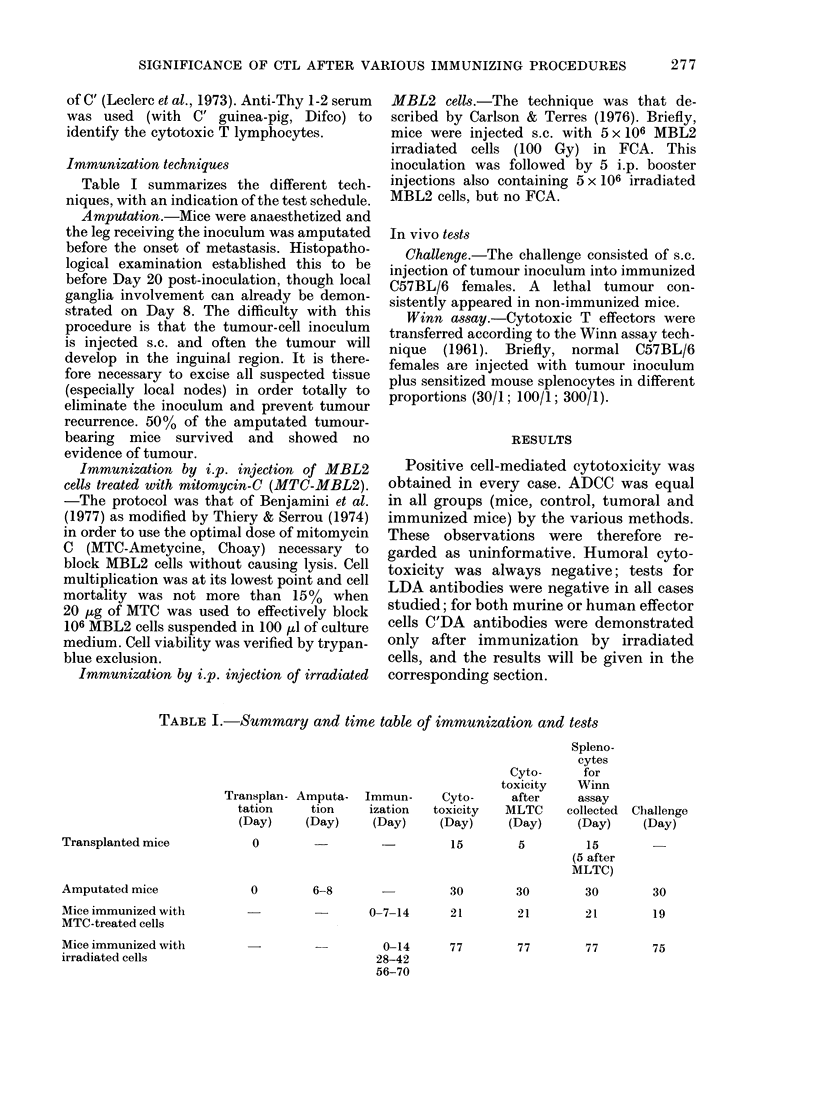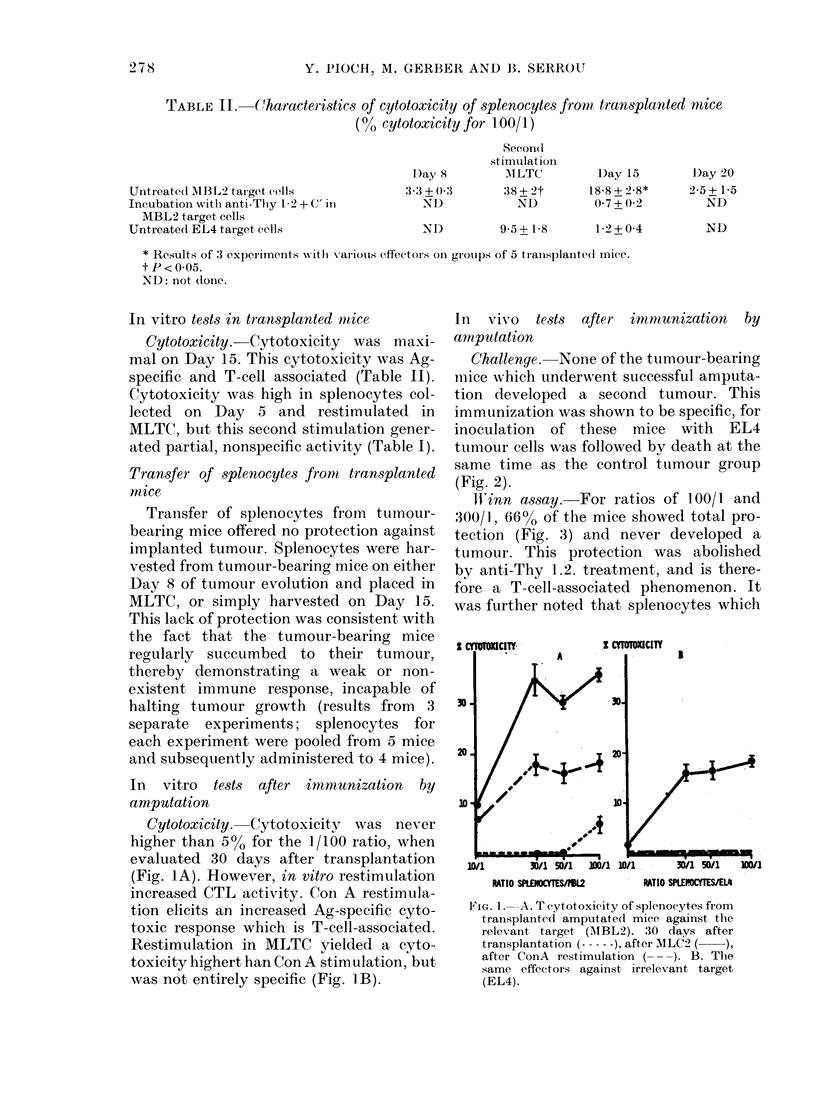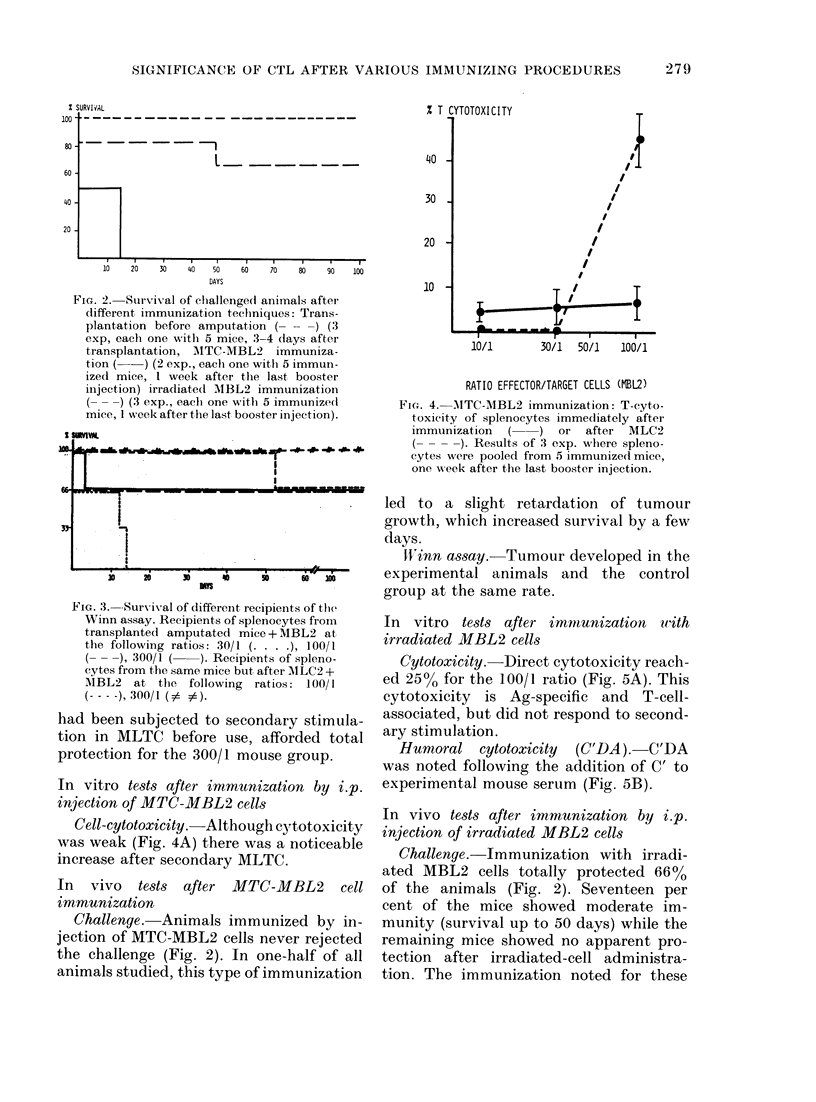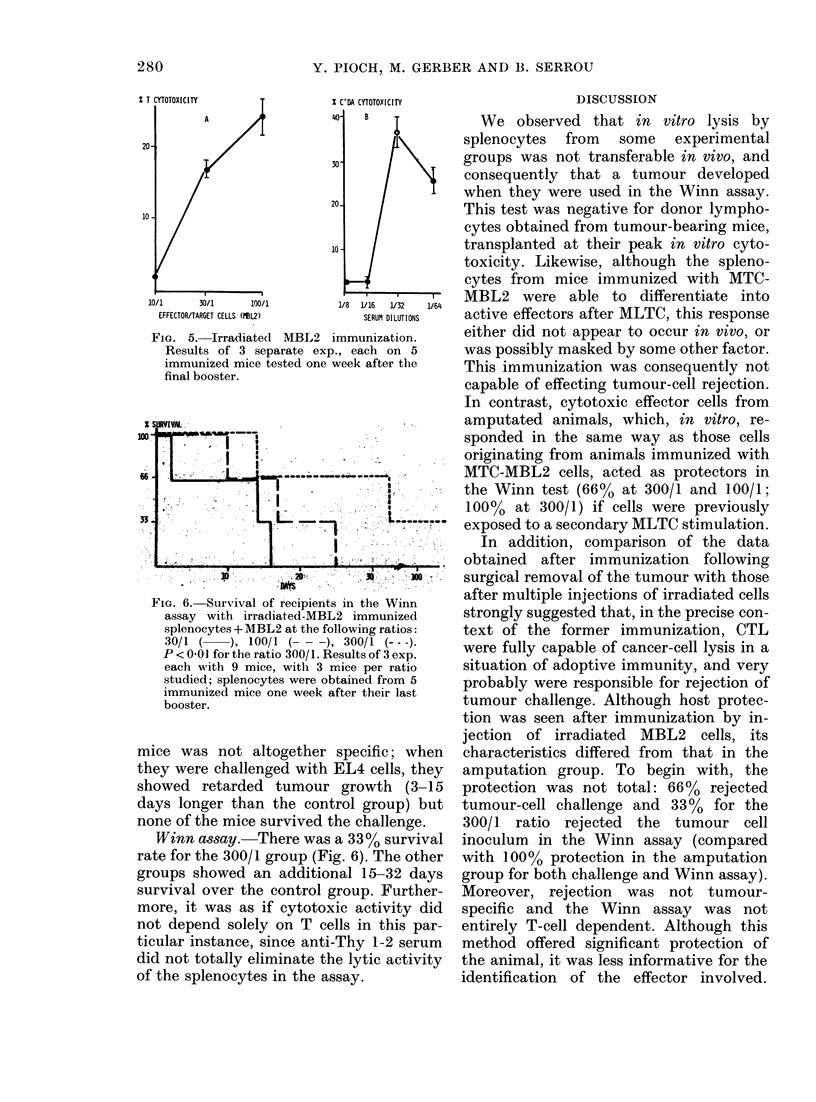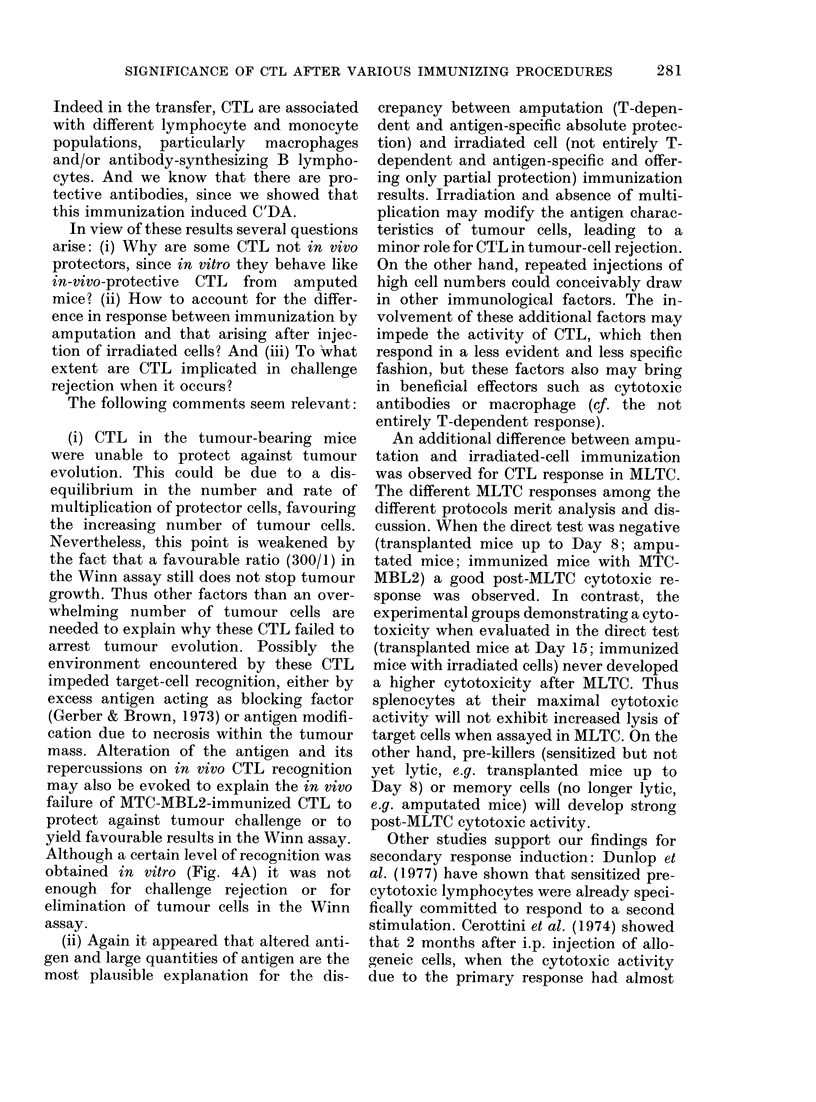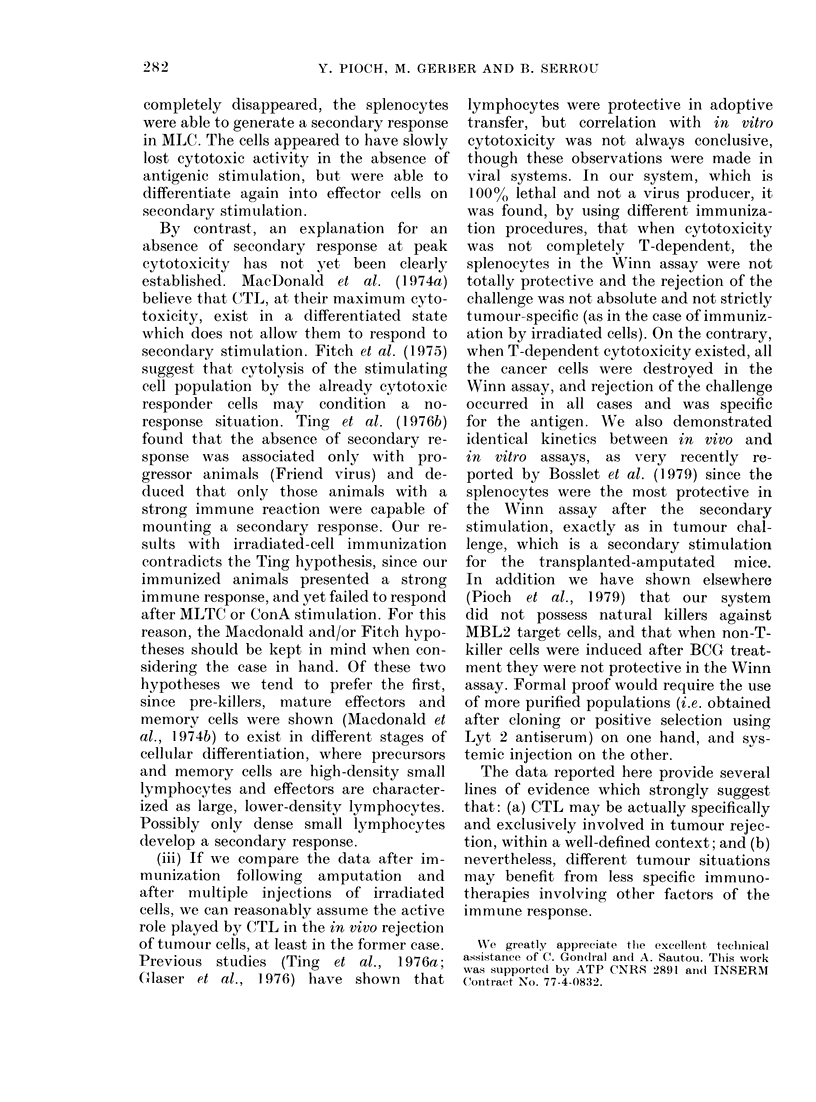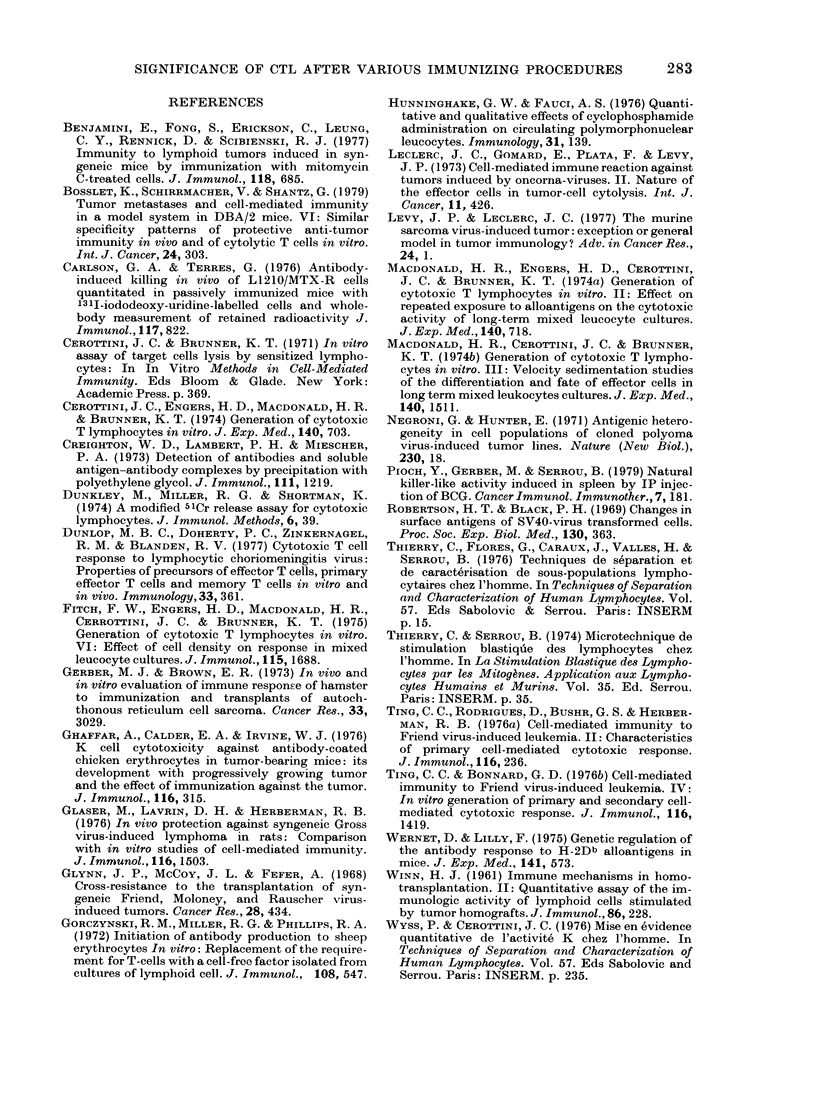# Significance of cytotoxic lymphocytes after various immunizing procedures in a virus-induced non-producer syngeneic system: correlation between in vitro and in vivo lytic activity.

**DOI:** 10.1038/bjc.1980.227

**Published:** 1980-08

**Authors:** Y. Pioch, M. Gerber, B. Serrou

## Abstract

An originally virus-induced, non-producer tumour system has been studied in relation to humoral and cellular cytotoxic responses to transplantation and other immunization techniques. In all experimental groups cytotoxic lymphocytes (CTL) were observed either directly or after mixed culture of lymphocytes and tumour cells (MLTC). Except for C'-dependent cytotoxic antibodies in mice immunized by irradiated cells, no antibody-mediated cytotoxicity was observed. In 2 protocols (transplantation and immunization by mitomycin-treated cells) CTL in vitro were not protective. In a third protocol (immunization by irradiated cells) CTL afforded partial protection and other factors appeared to be involved. The best in vivo protection was induced by immunization consequent on early surgical removal of a small number of transplanted tumour cells. This study provides lines of evidence for the effectiveness of protection supplied by CTL in well-defined conditions. Comparison with other modes of immunization indicated that these conditions were related to the quantity and to the characteristics of antigen involved.


Br. J. Cancer (1980) **42**, 275

### SIGNIFICANCE OF CYTOTOXIC LYMPHOCYTES AFTER VARIOUS IMMUNIZING PROCEDURES IN A VIRUS-INDUCED NON-PRODUCER SYNGENEIC SYSTEM: CORRELATION BETWEEN *IN VITRO* AND *IN VIVO* LYTIC ACTIVITY

Y. PIOCH, M. GERBER AND B. SERROU

*From the Laboratoire d'Immunopharmacologie des Tumeurs, Centre Paul Lamarque, Clinique St-Eloi, Montpellier, France*

Received 24 September 1979 Accepted 1 April 1980

**Summary.—An originally virus-induced, non-producer tumour system has been studied in relation to humoral and cellular cytotoxic responses to transplantation and other immunization techniques. In all experimental groups cytotoxic lymphocytes (CTL) were observed either directly or after mixed culture of lymphocytes and tumour cells (MLTC). Except for C′-dependent cytotoxic antibodies in mice immunized by irradiated cells, no antibody-mediated cytotoxicity was observed. In 2 protocols (transplantation and immunization by mitomycin-treated cells) CTL *in vitro* were not protective. In a third protocol (immunization by irradiated cells) CTL afforded partial protection and other factors appeared to be involved. The best *in vivo* protection was induced by immunization consequent on early surgical removal of a small number of transplanted tumour cells. This study provides lines of evidence for the effectiveness of protection supplied by CTL in well-defined conditions. Comparison with other modes of immunization indicated that these conditions were related to the quantity and to the characteristics of antigen involved.**

THE EXISTENCE of an immune response to tumour antigen has been demonstrated for several animal systems. This response is most often studied in terms of humoral parameters (Negroni & Hunter, 1971; Robertson & Black, 1969) but evidence for the presence of cytotoxic T lymphocytes (CTL) has given rise to a cellular approach to this same phenomenon. This approach has been particularly exploited in viral-murine systems (Levy & Leclerc, 1977) which have demonstrated raised T-cell cytotoxicity within a syngeneic context.

However, "little information is available that would allow one to determine whether the *in vitro* phenomena are relevant to *in vivo* tumour rejection" (Levy & Leclerc, 1977). The situation is even more obscure for transplantation of non-producer tumour cells. We have studied a model of this latter type, testing the *in vitro* activity of splenocytes and sera from transplanted mice which had never shown tumour rejection, as well as from mice immunized by different techniques. Correlation with *in vivo* protection was investigated by the Winn assay and the challenge inoculation of immunized mice.

We were able to show that, within a defined context, CTL were capable of tumour-cell lysis in adoptive transfer, and lines of evidence suggested that the CTL were responsible for tumour rejection in the challenged animal. But in other situations, *in-vitro*-effective CTL acted as non-protectors *in vivo*. Different responses were related to different modes of immunization. The best circumstance for an anti-tumour response was associated with early surgical removal of a small number of transplanted tumour cells. The unaltered limited amount of antigen appeared responsible for the induction of

Correspondence and reprints requests to Dr M. Gerber, Laboratoire d'Immunopharmacologie des Tumeurs, Centre Paul Lamarque, Clinique St-Eloi, B.P 5054 34033, Montpellier Cedex, France.

effective CTL, whereas transplantation without surgical removal led to overwhelming numbers of multiplying tumour cells, and immunization by irradiated cells led to a more complex and less protective response, possibly because of the repeated injection of modified antigen.

### MATERIALS AND METHODS

### *Experimental system*

The animals used in this study were 5–6-week-old C57BL/6 female mice. Our MBL2 tumour originated from a lymphosarcoma which was induced by Moloney virus (MSV) in C57BL/6 females (Glynn *et al.*, 1968). This line was maintained and continued in its ascitic form by i.p. injection of $10^6$ viable MBL2 cells. If $5 \times 10^4$ cells were injected s.c. into the rear thigh, a solid tumour appeared which was consistently lethal on Days 20–25 after inoculation.

In our laboratory, this line does not appear to be accompanied by viral propagation. This was ascertained after inoculating D56 ($S^+L^-$) cells with a cell filtrate from MSV-bearing MBL2 cells. Since the inoculation did not result in transformation of the D56 ($S^+L^-$) strain (Prof. C. Jasmin, ICIG, Villejuif, France) we concluded that the MBL2 cells do not support propagation of the virus. An EL4 tumour line derived from benzanthracene-induced lymphoma in C57BL/6 male mice were used to test the specificity of our results.

### In vitro *tests*

*T-type cytotoxicity*.—Target cells were MBL2 and effector cells were a total splenocyte population containing CTL. The technique has previously been described by Cerottini & Brunner (1971) and consists of incubating splenocytes at different ratios (10/1 to 100/1) with $10^4$ target cells labelled with chromium 51 ($^{51}$Cr-S1, Saclay). This mixture was incubated for 3 h at 37°C followed by 1 h at 45°C (Dunkley *et al.*, 1974). Results are expressed as percentage specific cytotoxicity ± s.d.

*Secondary MLTC-CML*.—Lymphocytes which had been sensitized *in vivo* were subsequently stimulated *in vitro* to produce CTL in mixed culture with tumour cells (MLTC). Cultures were carried out in 30ml flasks (25100 Corning CML) containing $40 \times 10^6$ splenocytes and $8 \times 10^6$ irradiated MBL2 cells (60 Gy, $^{60}$Co source, Gammatron III, Siemens) both of which are suspended in 20 ml of medium previously defined by Cerottini *et al.* (1974).

*Cytotoxic activity of killer cells* (*ADCC*).—Target cells were CRBC coated with rabbit anti-CRBC antibody. Effector cells were the entire splenocyte population. The technique was that of Ghaffar *et al.* (1976). Briefly, $10^4$-labelled CRBC were coated with anti-CRBC serum at selected dilution and incubated for 18 h at 37°C with splenic cells in variable proportions (10/1 to 100/1). Anti-serum was prepared using the Hunninghake & Fauci method (1976). Results are expressed as percentage specific cytotoxicity ± s.d.

*Lymphocyte-dependent antibody* (*LDA*).—Before undertaking this test, the absence of Fc receptor on MBL2 target cells was demonstrated by the EA-rosette technique which showed 2–3% rosettes (Thierry *et al.*, 1976).

The LDA technique is the same as that used to measure K-cell activity, but with the following modifications for effector and target cells: target MBL2 cells are coated with test serum at different dilutions (1/20 to 1/200). Mouse splenocytes or, even better, human lymphocytes (which are an excellent source of K cells, Wyss & Cerottini, 1976) were used as effector cells. We tried to eliminate false negative reactions due to Ag–Ab complexes by pre-incubating the test sera with polyethylene glycol (PEG) (Creighton *et al.*, 1973).

*Complement* (*C′*)*-dependent antibody* (*C′DA*).—MBL2 target cells were exposed to normal and experimental mouse serum plus C′ (guinea-pig, Gibco). The test procedure followed the technique described by Wernet & Lilly (1975). Briefly, $10^6$ $^{51}$Cr-labelled MBL2 cells were incubated at 37°C in test serum at different dilutions (1/4 to 1/640) in the presence of C′ for a predetermined time (1, 2 and 3 h). Sera were also tested after PEG incubation (Creighton *et al.*, 1973). Results are expressed as percentage specific cytotoxicity ± s.d.

*Anti-Thy* 1-2.—Anti-Thy 1-2 serum was prepared according to the technique of Gorczynski *et al.* (1972). Briefly, $10^8$ mouse (C3H) thymocytes in Freund's complete adjuvant (FCA, Difco) were injected into AKR mice. This procedure was repeated after 3 and 6 weeks. The specificity of the immune serum was verified using C57BL/6, BALB/c and AKR mouse thymocytes in the presence of C′ (Leclerc *et al.*, 1973). Anti-Thy 1-2 serum was used (with C′ guinea-pig, Difco) to identify the cytotoxic T lymphocytes.

*Immunization techniques*

Table I summarizes the different techniques, with an indication of the test schedule.

*Amputation.*—Mice were anaesthetized and the leg receiving the inoculum was amputated before the onset of metastasis. Histopathological examination established this to be before Day 20 post-inoculation, though local ganglia involvement can already be demonstrated on Day 8. The difficulty with this procedure is that the tumour-cell inoculum is injected s.c. and often the tumour will develop in the inguinal region. It is therefore necessary to excise all suspected tissue (especially local nodes) in order totally to eliminate the inoculum and prevent tumour recurrence. 50% of the amputated tumour-bearing mice survived and showed no evidence of tumour.

*Immunization by i.p. injection of MBL2 cells treated with mitomycin-C (MTC-MBL2).*—The protocol was that of Benjamini *et al.* (1977) as modified by Thiery & Serrou (1974) in order to use the optimal dose of mitomycin C (MTC-Ametycine, Choay) necessary to block MBL2 cells without causing lysis. Cell multiplication was at its lowest point and cell mortality was not more than 15% when 20 μg of MTC was used to effectively block $10^6$ MBL2 cells suspended in 100 μl of culture medium. Cell viability was verified by trypan-blue exclusion.

*Immunization by i.p. injection of irradiated MBL2 cells.*—The technique was that described by Carlson & Terres (1976). Briefly, mice were injected s.c. with $5 \times 10^6$ MBL2 irradiated cells (100 Gy) in FCA. This inoculation was followed by 5 i.p. booster injections also containing $5 \times 10^6$ irradiated MBL2 cells, but no FCA.

In vivo *tests*

*Challenge.*—The challenge consisted of s.c. injection of tumour inoculum into immunized C57BL/6 females. A lethal tumour consistently appeared in non-immunized mice.

*Winn assay.*—Cytotoxic T effectors were transferred according to the Winn assay technique (1961). Briefly, normal C57BL/6 females are injected with tumour inoculum plus sensitized mouse splenocytes in different proportions (30/1; 100/1; 300/1).

### RESULTS

Positive cell-mediated cytotoxicity was obtained in every case. ADCC was equal in all groups (mice, control, tumoral and immunized mice) by the various methods. These observations were therefore regarded as uninformative. Humoral cytotoxicity was always negative; tests for LDA antibodies were negative in all cases studied; for both murine or human effector cells C′DA antibodies were demonstrated only after immunization by irradiated cells, and the results will be given in the corresponding section.

TABLE I.—*Summary and time table of immunization and tests*

| | Transplantation (Day) | Amputation (Day) | Immunization (Day) | Cytotoxicity (Day) | Cytotoxicity after MLTC (Day) | Splenocytes for Winn assay collected (Day) | Challenge (Day) |
|---|---|---|---|---|---|---|---|
| Transplanted mice | 0 | — | — | 15 | 5 | 15 (5 after MLTC) | — |
| Amputated mice | 0 | 6–8 | — | 30 | 30 | 30 | 30 |
| Mice immunized with MTC-treated cells | — | — | 0–7–14 | 21 | 21 | 21 | 19 |
| Mice immunized with irradiated cells | — | — | 0–14 28–42 56–70 | 77 | 77 | 77 | 75 |

TABLE II.—*Characteristics of cytotoxicity of splenocytes from transplanted mice (% cytotoxicity for* 100/1)

| | Day 8 | Second stimulation MLTC | Day 15 | Day 20 |
|---|---|---|---|---|
| Untreated MBL2 target cells | 3·3 ± 0·3 | 38 ± 2† | 18·8 ± 2·8* | 2·5 ± 1·5 |
| Incubation with anti-Thy 1·2 + C′ in MBL2 target cells | ND | ND | 0·7 ± 0·2 | ND |
| Untreated EL4 target cells | ND | 9·5 ± 1·8 | 1·2 ± 0·4 | ND |

* Results of 3 experiments with various effectors on groups of 5 transplanted mice.

† $P < 0·05$.

ND: not done.

In vitro *tests in transplanted mice*

*Cytotoxicity.*—Cytotoxicity was maximal on Day 15. This cytotoxicity was Ag-specific and T-cell associated (Table II). Cytotoxicity was high in splenocytes collected on Day 5 and restimulated in MLTC, but this second stimulation generated partial, nonspecific activity (Table I).

*Transfer of splenocytes from transplanted mice*

Transfer of splenocytes from tumour-bearing mice offered no protection against implanted tumour. Splenocytes were harvested from tumour-bearing mice on either Day 8 of tumour evolution and placed in MLTC, or simply harvested on Day 15. This lack of protection was consistent with the fact that the tumour-bearing mice regularly succumbed to their tumour, thereby demonstrating a weak or non-existent immune response, incapable of halting tumour growth (results from 3 separate experiments; splenocytes for each experiment were pooled from 5 mice and subsequently administered to 4 mice).

In vitro *tests after immunization by amputation*

*Cytotoxicity.*—Cytotoxicity was never higher than 5% for the 1/100 ratio, when evaluated 30 days after transplantation (Fig. 1A). However, *in vitro* restimulation increased CTL activity. Con A restimulation elicits an increased Ag-specific cytotoxic response which is T-cell-associated. Restimulation in MLTC yielded a cytotoxicity highert han Con A stimulation, but was not entirely specific (Fig. 1B).

In vivo *tests after immunization by amputation*

*Challenge.*—None of the tumour-bearing mice which underwent successful amputation developed a second tumour. This immunization was shown to be specific, for inoculation of these mice with EL4 tumour cells was followed by death at the same time as the control tumour group (Fig. 2).

*Winn assay.*—For ratios of 100/1 and 300/1, 66% of the mice showed total protection (Fig. 3) and never developed a tumour. This protection was abolished by anti-Thy 1.2. treatment, and is therefore a T-cell-associated phenomenon. It was further noted that splenocytes which

FIG. 1.—A. T cytotoxicity of splenocytes from transplanted amputated mice against the relevant target (MBL2). 30 days after transplantation (- - - - -), after MLC2 (——), after ConA restimulation (– – –). B. The same effectors against irrelevant target (EL4).

FIG. 2.—Survival of challenged animals after different immunization techniques: Transplantation before amputation (– – –) (3 exp, each one with 5 mice, 3–4 days after transplantation, MTC-MBL2 immunization (——) (2 exp., each one with 5 immunized mice, 1 week after the last booster injection) irradiated MBL2 immunization (– – –) (3 exp., each one with 5 immunized mice, 1 week after the last booster injection).

FIG. 3.—Survival of different recipients of the Winn assay. Recipients of splenocytes from transplanted amputated mice+MBL2 at the following ratios: 30/1 (. . . .), 100/1 (– – –), 300/1 (——). Recipients of splenocytes from the same mice but after MLC2+MBL2 at the following ratios: 100/1 (- - - -), 300/1 (≠ ≠).

had been subjected to secondary stimulation in MLTC before use, afforded total protection for the 300/1 mouse group.

### In vitro *tests after immunization by i.p. injection of MTC-MBL2 cells*

*Cell-cytotoxicity.*—Although cytotoxicity was weak (Fig. 4A) there was a noticeable increase after secondary MLTC.

### In vivo *tests after MTC-MBL2 cell immunization*

*Challenge.*—Animals immunized by injection of MTC-MBL2 cells never rejected the challenge (Fig. 2). In one-half of all animals studied, this type of immunization

FIG. 4.—MTC-MBL2 immunization: T-cytotoxicity of splenocytes immediately after immunization (——) or after MLC2 (– – – –). Results of 3 exp. where splenocytes were pooled from 5 immunized mice, one week after the last booster injection.

led to a slight retardation of tumour growth, which increased survival by a few days.

*Winn assay.*—Tumour developed in the experimental animals and the control group at the same rate.

### In vitro *tests after immunization with irradiated MBL2 cells*

*Cytotoxicity.*—Direct cytotoxicity reached 25% for the 100/1 ratio (Fig. 5A). This cytotoxicity is Ag-specific and T-cell-associated, but did not respond to secondary stimulation.

*Humoral cytotoxicity (C′DA).*—C′DA was noted following the addition of C′ to experimental mouse serum (Fig. 5B).

### In vivo *tests after immunization by i.p. injection of irradiated MBL2 cells*

*Challenge.*—Immunization with irradiated MBL2 cells totally protected 66% of the animals (Fig. 2). Seventeen per cent of the mice showed moderate immunity (survival up to 50 days) while the remaining mice showed no apparent protection after irradiated-cell administration. The immunization noted for these

FIG. 5.—Irradiated MBL2 immunization. Results of 3 separate exp., each on 5 immunized mice tested one week after the final booster.

FIG. 6.—Survival of recipients in the Winn assay with irradiated-MBL2 immunized splenocytes + MBL2 at the following ratios: 30/1 (——), 100/1 (– – –), 300/1 (- - -). $P < 0{\cdot}01$ for the ratio 300/1. Results of 3 exp. each with 9 mice, with 3 mice per ratio studied; splenocytes were obtained from 5 immunized mice one week after their last booster.

mice was not altogether specific; when they were challenged with EL4 cells, they showed retarded tumour growth (3–15 days longer than the control group) but none of the mice survived the challenge.

*Winn assay.*—There was a 33% survival rate for the 300/1 group (Fig. 6). The other groups showed an additional 15–32 days survival over the control group. Furthermore, it was as if cytotoxic activity did not depend solely on T cells in this particular instance, since anti-Thy 1-2 serum did not totally eliminate the lytic activity of the splenocytes in the assay.

### DISCUSSION

We observed that *in vitro* lysis by splenocytes from some experimental groups was not transferable *in vivo*, and consequently that a tumour developed when they were used in the Winn assay. This test was negative for donor lymphocytes obtained from tumour-bearing mice, transplanted at their peak *in vitro* cytotoxicity. Likewise, although the splenocytes from mice immunized with MTC-MBL2 were able to differentiate into active effectors after MLTC, this response either did not appear to occur *in vivo*, or was possibly masked by some other factor. This immunization was consequently not capable of effecting tumour-cell rejection. In contrast, cytotoxic effector cells from amputated animals, which, *in vitro*, responded in the same way as those cells originating from animals immunized with MTC-MBL2 cells, acted as protectors in the Winn test (66% at 300/1 and 100/1; 100% at 300/1) if cells were previously exposed to a secondary MLTC stimulation.

In addition, comparison of the data obtained after immunization following surgical removal of the tumour with those after multiple injections of irradiated cells strongly suggested that, in the precise context of the former immunization, CTL were fully capable of cancer-cell lysis in a situation of adoptive immunity, and very probably were responsible for rejection of tumour challenge. Although host protection was seen after immunization by injection of irradiated MBL2 cells, its characteristics differed from that in the amputation group. To begin with, the protection was not total: 66% rejected tumour-cell challenge and 33% for the 300/1 ratio rejected the tumour cell inoculum in the Winn assay (compared with 100% protection in the amputation group for both challenge and Winn assay). Moreover, rejection was not tumour-specific and the Winn assay was not entirely T-cell dependent. Although this method offered significant protection of the animal, it was less informative for the identification of the effector involved.

Indeed in the transfer, CTL are associated with different lymphocyte and monocyte populations, particularly macrophages and/or antibody-synthesizing B lymphocytes. And we know that there are protective antibodies, since we showed that this immunization induced C′DA.

In view of these results several questions arise: (i) Why are some CTL not *in vivo* protectors, since *in vitro* they behave like *in-vivo*-protective CTL from amputed mice? (ii) How to account for the difference in response between immunization by amputation and that arising after injection of irradiated cells? And (iii) To what extent are CTL implicated in challenge rejection when it occurs?

The following comments seem relevant:

(i) CTL in the tumour-bearing mice were unable to protect against tumour evolution. This could be due to a disequilibrium in the number and rate of multiplication of protector cells, favouring the increasing number of tumour cells. Nevertheless, this point is weakened by the fact that a favourable ratio (300/1) in the Winn assay still does not stop tumour growth. Thus other factors than an overwhelming number of tumour cells are needed to explain why these CTL failed to arrest tumour evolution. Possibly the environment encountered by these CTL impeded target-cell recognition, either by excess antigen acting as blocking factor (Gerber & Brown, 1973) or antigen modification due to necrosis within the tumour mass. Alteration of the antigen and its repercussions on *in vivo* CTL recognition may also be evoked to explain the *in vivo* failure of MTC-MBL2-immunized CTL to protect against tumour challenge or to yield favourable results in the Winn assay. Although a certain level of recognition was obtained *in vitro* (Fig. 4A) it was not enough for challenge rejection or for elimination of tumour cells in the Winn assay.

(ii) Again it appeared that altered antigen and large quantities of antigen are the most plausible explanation for the discrepancy between amputation (T-dependent and antigen-specific absolute protection) and irradiated cell (not entirely T-dependent and antigen-specific and offering only partial protection) immunization results. Irradiation and absence of multiplication may modify the antigen characteristics of tumour cells, leading to a minor role for CTL in tumour-cell rejection. On the other hand, repeated injections of high cell numbers could conceivably draw in other immunological factors. The involvement of these additional factors may impede the activity of CTL, which then respond in a less evident and less specific fashion, but these factors also may bring in beneficial effectors such as cytotoxic antibodies or macrophage (*cf.* the not entirely T-dependent response).

An additional difference between amputation and irradiated-cell immunization was observed for CTL response in MLTC. The different MLTC responses among the different protocols merit analysis and discussion. When the direct test was negative (transplanted mice up to Day 8; amputated mice; immunized mice with MTC-MBL2) a good post-MLTC cytotoxic response was observed. In contrast, the experimental groups demonstrating a cytotoxicity when evaluated in the direct test (transplanted mice at Day 15; immunized mice with irradiated cells) never developed a higher cytotoxicity after MLTC. Thus splenocytes at their maximal cytotoxic activity will not exhibit increased lysis of target cells when assayed in MLTC. On the other hand, pre-killers (sensitized but not yet lytic, *e.g.* transplanted mice up to Day 8) or memory cells (no longer lytic, *e.g.* amputated mice) will develop strong post-MLTC cytotoxic activity.

Other studies support our findings for secondary response induction: Dunlop *et al.* (1977) have shown that sensitized precytotoxic lymphocytes were already specifically committed to respond to a second stimulation. Cerottini *et al.* (1974) showed that 2 months after i.p. injection of allogeneic cells, when the cytotoxic activity due to the primary response had almost completely disappeared, the splenocytes were able to generate a secondary response in MLC. The cells appeared to have slowly lost cytotoxic activity in the absence of antigenic stimulation, but were able to differentiate again into effector cells on secondary stimulation.

By contrast, an explanation for an absence of secondary response at peak cytotoxicity has not yet been clearly established. MacDonald *et al.* (1974*a*) believe that CTL, at their maximum cytotoxicity, exist in a differentiated state which does not allow them to respond to secondary stimulation. Fitch *et al.* (1975) suggest that cytolysis of the stimulating cell population by the already cytotoxic responder cells may condition a no-response situation. Ting *et al.* (1976*b*) found that the absence of secondary response was associated only with progressor animals (Friend virus) and deduced that only those animals with a strong immune reaction were capable of mounting a secondary response. Our results with irradiated-cell immunization contradicts the Ting hypothesis, since our immunized animals presented a strong immune response, and yet failed to respond after MLTC or ConA stimulation. For this reason, the Macdonald and/or Fitch hypotheses should be kept in mind when considering the case in hand. Of these two hypotheses we tend to prefer the first, since pre-killers, mature effectors and memory cells were shown (Macdonald *et al.*, 1974*b*) to exist in different stages of cellular differentiation, where precursors and memory cells are high-density small lymphocytes and effectors are characterized as large, lower-density lymphocytes. Possibly only dense small lymphocytes develop a secondary response.

(iii) If we compare the data after immunization following amputation and after multiple injections of irradiated cells, we can reasonably assume the active role played by CTL in the *in vivo* rejection of tumour cells, at least in the former case. Previous studies (Ting *et al.*, 1976*a*; Glaser *et al.*, 1976) have shown that lymphocytes were protective in adoptive transfer, but correlation with *in vitro* cytotoxicity was not always conclusive, though these observations were made in viral systems. In our system, which is 100% lethal and not a virus producer, it was found, by using different immunization procedures, that when cytotoxicity was not completely T-dependent, the splenocytes in the Winn assay were not totally protective and the rejection of the challenge was not absolute and not strictly tumour-specific (as in the case of immunization by irradiated cells). On the contrary, when T-dependent cytotoxicity existed, all the cancer cells were destroyed in the Winn assay, and rejection of the challenge occurred in all cases and was specific for the antigen. We also demonstrated identical kinetics between *in vivo* and *in vitro* assays, as very recently reported by Bosslet *et al.* (1979) since the splenocytes were the most protective in the Winn assay after the secondary stimulation, exactly as in tumour challenge, which is a secondary stimulation for the transplanted-amputated mice. In addition we have shown elsewhere (Pioch *et al.*, 1979) that our system did not possess natural killers against MBL2 target cells, and that when non-T-killer cells were induced after BCG treatment they were not protective in the Winn assay. Formal proof would require the use of more purified populations (*i.e.* obtained after cloning or positive selection using Lyt 2 antiserum) on one hand, and systemic injection on the other.

The data reported here provide several lines of evidence which strongly suggest that: (a) CTL may be actually specifically and exclusively involved in tumour rejection, within a well-defined context; and (b) nevertheless, different tumour situations may benefit from less specific immunotherapies involving other factors of the immune response.

We greatly appreciate the excellent technical assistance of C. Gondral and A. Sautou. This work was supported by ATP CNRS 2891 and INSERM Contract No. 77-4-0832.

### REFERENCES

BENJAMINI, E., FONG, S., ERICKSON, C., LEUNG, C. Y., RENNICK, D. & SCIBIENSKI, R. J. (1977) Immunity to lymphoid tumors induced in syngeneic mice by immunization with mitomycin C-treated cells. *J. Immunol.*, **118**, 685.

BOSSLET, K., SCHIRRMACHER, V. & SHANTZ, G. (1979) Tumor metastases and cell-mediated immunity in a model system in DBA/2 mice. VI: Similar specificity patterns of protective anti-tumor immunity *in vivo* and of cytolytic T cells *in vitro*. *Int. J. Cancer*, **24**, 303.

CARLSON, G. A. & TERRES, G. (1976) Antibody-induced killing *in vivo* of L1210/MTX-R cells quantitated in passively immunized mice with $^{131}$I-iododeoxy-uridine-labelled cells and whole-body measurement of retained radioactivity *J. Immunol.*, **117**, 822.

CEROTTINI, J. C. & BRUNNER, K. T. (1971) *In vitro* assay of target cells lysis by sensitized lymphocytes: In In Vitro *Methods in Cell-Mediated Immunity*. Eds Bloom & Glade. New York: Academic Press. p. 369.

CEROTTINI, J. C., ENGERS, H. D., MACDONALD, H. R. & BRUNNER, K. T. (1974) Generation of cytotoxic T lymphocytes *in vitro*. *J. Exp. Med.*, **140**, 703.

CREIGHTON, W. D., LAMBERT, P. H. & MIESCHER, P. A. (1973) Detection of antibodies and soluble antigen–antibody complexes by precipitation with polyethylene glycol. *J. Immunol.*, **111**, 1219.

DUNKLEY, M., MILLER, R. G. & SHORTMAN, K. (1974) A modified $^{51}$Cr release assay for cytotoxic lymphocytes. *J. Immunol. Methods*, **6**, 39.

DUNLOP, M. B. C., DOHERTY, P. C., ZINKERNAGEL, R. M. & BLANDEN, R. V. (1977) Cytotoxic T cell response to lymphocytic choriomeningitis virus: Properties of precursors of effector T cells, primary effector T cells and memory T cells *in vitro* and *in vivo*. *Immunology*, **33**, 361.

FITCH, F. W., ENGERS, H. D., MACDONALD, H. R., CERROTTINI, J. C. & BRUNNER, K. T. (1975) Generation of cytotoxic T lymphocytes *in vitro*. VI: Effect of cell density on response in mixed leucocyte cultures. *J. Immunol.*, **115**, 1688.

GERBER, M. J. & BROWN, E. R. (1973) *In vivo* and *in vitro* evaluation of immune response of hamster to immunization and transplants of autochthonous reticulum cell sarcoma. *Cancer Res.*, **33**, 3029.

GHAFFAR, A., CALDER, E. A. & IRVINE, W. J. (1976) K cell cytotoxicity against antibody-coated chicken erythrocytes in tumor-bearing mice: its development with progressively growing tumor and the effect of immunization against the tumor. *J. Immunol.*, **116**, 315.

GLASER, M., LAVRIN, D. H. & HERBERMAN, R. B. (1976) *In vivo* protection against syngeneic Gross virus-induced lymphoma in rats: Comparison with *in vitro* studies of cell-mediated immunity. *J. Immunol.*, **116**, 1503.

GLYNN, J. P., MCCOY, J. L. & FEFER, A. (1968) Cross-resistance to the transplantation of syngeneic Friend, Moloney, and Rauscher virus-induced tumors. *Cancer Res.*, **28**, 434.

GORCZYNSKI, R. M., MILLER, R. G. & PHILLIPS, R. A. (1972) Initiation of antibody production to sheep erythrocytes *In vitro*: Replacement of the requirement for T-cells with a cell-free factor isolated from cultures of lymphoid cell. *J. Immunol.*, **108**, 547.

HUNNINGHAKE, G. W. & FAUCI, A. S. (1976) Quantitative and qualitative effects of cyclophosphamide administration on circulating polymorphonuclear leucocytes. *Immunology*, **31**, 139.

LECLERC, J. C., GOMARD, E., PLATA, F. & LEVY, J. P. (1973) Cell-mediated immune reaction against tumors induced by oncorna-viruses. II. Nature of the effector cells in tumor-cell cytolysis. *Int. J. Cancer*, **11**, 426.

LEVY, J. P. & LECLERC, J. C. (1977) The murine sarcoma virus-induced tumor: exception or general model in tumor immunology? *Adv. in Cancer Res.*, **24**, 1.

MACDONALD, H. R., ENGERS, H. D., CEROTTINI, J. C. & BRUNNER, K. T. (1974*a*) Generation of cytotoxic T lymphocytes *in vitro*. II: Effect on repeated exposure to alloantigens on the cytotoxic activity of long-term mixed leucocyte cultures. *J. Exp. Med.*, **140**, 718.

MACDONALD, H. R., CEROTTINI, J. C. & BRUNNER, K. T. (1974*b*) Generation of cytotoxic T lymphocytes *in vitro*. III: Velocity sedimentation studies of the differentiation and fate of effector cells in long term mixed leukocytes cultures. *J. Exp. Med.*, **140**, 1511.

NEGRONI, G. & HUNTER, E. (1971) Antigenic heterogeneity in cell populations of cloned polyoma virus-induced tumor lines. *Nature* (*New Biol.*), **230**, 18.

PIOCH, Y., GERBER, M. & SERROU, B. (1979) Natural killer-like activity induced in spleen by IP injection of BCG. *Cancer Immunol. Immunother.*, **7**, 181.

ROBERTSON, H. T. & BLACK, P. H. (1969) Changes in surface antigens of SV40-virus transformed cells. *Proc. Soc. Exp. Biol. Med.*, **130**, 363.

THIERRY, C., FLORES, G., CARAUX, J., VALLES, H. & SERROU, B. (1976) Techniques de séparation et de caractérisation de sous-populations lymphocytaires chez l'homme. In *Techniques of Separation and Characterization of Human Lymphocytes*. Vol. 57. Eds Sabolovic & Serrou. Paris: INSERM p. 15.

THIERRY, C. & SERROU, B. (1974) Microtechnique de stimulation blastiqúe des lymphocytes chez l'homme. In *La Stimulation Blastique des Lymphocytes par les Mitogènes. Application aux Lymphocytes Humains et Murins*. Vol. 35. Ed. Serrou. Paris: INSERM. p. 35.

TING, C. C., RODRIGUES, D., BUSHR, G. S. & HERBERMAN, R. B. (1976*a*) Cell-mediated immunity to Friend virus-induced leukemia. II: Characteristics of primary cell-mediated cytotoxic response. *J. Immunol.*, **116**, 236.

TING, C. C. & BONNARD, G. D. (1976*b*) Cell-mediated immunity to Friend virus-induced leukemia. IV: *In vitro* generation of primary and secondary cell-mediated cytotoxic response. *J. Immunol.*, **116**, 1419.

WERNET, D. & LILLY, F. (1975) Genetic regulation of the antibody response to H-2D$^b$ alloantigens in mice. *J. Exp. Med.*, **141**, 573.

WINN, H. J. (1961) Immune mechanisms in homotransplantation. II: Quantitative assay of the immunologic activity of lymphoid cells stimulated by tumor homografts. *J. Immunol.*, **86**, 228.

WYSS, P. & CEROTTINI, J. C. (1976) Mise en évidence quantitative de l'activité K chez l'homme. In *Techniques of Separation and Characterization of Human Lymphocytes*. Vol. 57. Eds Sabolovic and Serrou. Paris: INSERM. p. 235.